# Ion channel function in translational bovine gallbladder cholangiocyte organoids: establishment and characterization of a novel model system

**DOI:** 10.3389/fvets.2023.1179836

**Published:** 2023-05-26

**Authors:** Itsuma Nagao, Yoko M. Ambrosini

**Affiliations:** ^1^Department of Veterinary Clinical Sciences, College of Veterinary Medicine, Washington State University, Pullman, WA, United States; ^2^Department of Veterinary Internal Medicine, Graduate School of Agricultural and Life Sciences, The University of Tokyo, Bunkyo, Tokyo, Japan

**Keywords:** bovine, gallbladder, organoids, cystic fibrosis transmembrane conductance regulator, translational medicine, comparative medicine

## Abstract

The study of biliary physiology and pathophysiology has long been hindered by the lack of *in vitro* models that accurately reflect the complex functions of the biliary system. Recent advancements in 3D organoid technology may offer a promising solution to this issue. Bovine gallbladder models have recently gained attention in the investigation of human diseases due to their remarkable similarities in physiology and pathophysiology with the human gallbladder. In this study, we have successfully established and characterized bovine gallbladder cholangiocyte organoids (GCOs) that retain key characteristics of the gallbladder *in vivo*, including stem cell properties and proliferative capacity. Notably, our findings demonstrate that these organoids exhibit specific and functional CFTR activity. We believe that these bovine GCOs represent a valuable tool for studying the physiology and pathophysiology of the gallbladder with human significance.

## 1. Introduction

The gallbladder stores and releases bile, which is essential for the digestion of fats (1). Abnormalities in gallbladder function and structure can lead to a range of disorders, including gallstones, cholecystitis, and gallbladder cancer (2). The study of biliary physiology has been hampered by the lack of *in vitro* models that accurately represent the complex functions of the biliary system (3). However, recent advancements in organoid technology have provided a promising breakthrough in this field (4, 5). Organoids are three-dimensional (3D) culture technologies that can recapitulate the architecture and function of donor tissues, closely mimicking the characteristics of donor epithelial cells, including gene expression patterns and structural features (6–9). Organoids, derived from stem cells, offer a unique platform for mimicking the complexity of living tissues, as they contain various cell types and recreate the stem cell niche *in vivo* (4, 6, 7, 9). Additionally, numerous studies have demonstrated that organoids exhibit gene expression patterns that closely resemble those found in living organs (1–4), and these patterns remain stable over multiple passages (9, 10). These similarities between organoids and *in vivo* organs suggest that organoids can accurately reflect the characteristics of the donor tissue. This allows the investigation of cellular behavior and physiological processes in a more accurate and physiologically relevant manner, providing greater insights into the underlying mechanisms of various biliary disorders and potential therapeutic interventions.

Bovine gallbladders can be accessibly obtained from healthy animals, whereas human gallbladders are often obtained from patients who have undergone surgical procedures due to gallbladder-related diseases (11, 12). This can make it difficult to expand normal human gallbladder tissue *in vitro* for research purposes. By using bovine gallbladders as a model for human gallbladder physiology, the ion transport properties of the organ without the ethical and practical concerns associated with obtaining normal human tissue samples can be accomplished. The close resemblance between the mechanisms of electrolyte transport and hydrogen ion secretion in the gallbladder epithelium of bovine and human species is highly relevant in the study of potential translational applications of bovine gallbladders (13, 14). Furthermore, bovine bile has been shown to have lithogenic properties, which may have implications in the study of gallstone formation and treatment (15–18).

Furthermore, isolation and persistent colonization of *E. coli* O157:H7 and *Salmonella* in the gallbladders of cattle have been investigated (19, 20) and these facts pose a risk to humans who come into contact with contaminated animal products (21, 22). Therefore, investigating the mechanisms by which these pathogens colonize and persist in the bovine gallbladder could lead to the development of more effective control measures to reduce the risk of human exposure to these pathogens.

In this study, we established organoids derived from bovine normal gallbladder from both calf and adult cattle. The morphology, protein expression, and proliferation were analyzed. Finally, we investigated the function of cystic fibrosis transmembrane conductance regulator (CFTR), one of the ion channels that is mainly expressed in epithelial cells, in bovine GCOs. Our main objective of this study was to demonstrate a proof-of-concept study where the reliable production of GCOs from bovine tissue and the feasibility of performing well-established ion transport assays in this *in vitro* system.

## 2. Materials and methods

### 2.1. Bovine gallbladder tissue acquisition

The gallbladder tissues from calves and beef yearling were collected from local slaughterhouses for this study. All donors in our study were clinically healthy cows with normal gallbladders since they were free of digestive and biliary symptoms, and we observed no edema, hemorrhage, parasites, or obvious inflammation on the mucosal surface of the gallbladder tissues. The animals used in this study were humanely euthanized by gunshot, which is a widely accepted method for rapid and humane slaughter on farms in North America. Following euthanasia, blood was collected *via* the carotid artery to avoid any potential confounding effects of stress or other factors associated with prolonged exsanguination (23). Briefly, immediately following the slaughtering, the gallbladder was isolated using sterile surgical scissors and opened longitudinally. Half of the gallbladder was put in ice-cold Dulbecco’s phosphate-buffered saline (PBS, Gibco) supplemented with 1x Penicillin/Streptomycin (Gibco), and 25 μg/mL gentamicin (Gibco) and kept on ice until further processing. All procedures associated with tissue sampling were exempt by the Washington State University Institutional Animal Care and Use Committee (IACUC) because the gallbladder samples were harvested from animals after they were humanely euthanized or slaughtered for purposes unrelated to this project.

### 2.2. Development of bovine GCO model

Methodology for the organoid model was adapted from published protocols for human gallbladder tissues (11). Tissue samples were rinsed five times with sterile PBS supplemented with penicillin, streptomycin, and gentamicin. The tissues were incubated in 0.25% w/v% Trypsin (Gibco) at 37°C for 60 min. After the inactivation of trypsin by adding 10% fetal bovine serum (FBS, Gibco), allow the tissues to settle, then collect the upper portion of the fluid containing free stem cells. The collected portion was filtered through a 100 μm cell strainer (Fisher Scientific), and centrifuged at 200×*g*, 4°C for 5 min. After another wash with DMEM/F12 (Gibco) supplemented with 1x Penicillin/Streptomycin (Gibco), 2 mM GultaMAX (Gibco), and 10 mM HEPES (Gibco), the pellet was resuspended in Matrigel (Corning) and seeded to a 24-well plate (Thermo Scientific) in 30 μL per well. After the seeding, the plate was inverted and incubated at 37°C for 10–15 min until the Matrigel dome was solidified. Then, 500 μL of culture medium was added to each well. Composition of the GCO culture medium was adapted from published protocols for human gallbladder tissues (11) (Supplementary Table S1). Briefly, DMEM/F12 (Gibco) was supplemented with 2 mM GultaMAX (Gibco), 10 mM HEPES (Gibco), 1x Penicillin/Streptomycin (Gibco), 10%(vol/vol) conditioned medium of Noggin (24), 20%(vol/vol) conditioned medium of R-spondin, 100 ng/mL recombinant murine Wnt-3a (PeproTech), 50 ng/mL murine Epidermal Growth Factor (EGF) (R&D Systems), 100 ng/mL human Fibroblast Growth Factor-10 (FGF10) (PeproTech), 25 ng/mL Hepatocyte Growth Factor (HGF) (PeproTech), 10 nM Gastrin (Sigma-Aldrich), 500 nM A-83-01 (Sigma-Aldrich), 10 μM SB202190 (Sigma-Aldrich), 1 mM N-Acetyl-L-cysteine (MP Biomedicals), 10 mM Nicotinamide (Sigma-Aldrich), 1x B27 supplement (Gibco), 1x N2 MAX Media supplement (R&D Systems), 100 μg/mL Primocin (InvivoGen), 10 μM Y-27632 (StemCellTechnologies), and 100 nM Dexamethasone (R&D Systems). The culture medium was replaced every other day. Organoids were passaged approximately once every 1–2 weeks. Matrigel dome was dissociated by incubating it with Cell Recovery Solution (Corning) for 30 min at 4°C. After collection of organoids containing solution, organoids were broken down using TrypLE Express (Gibco). Following 1 min incubation at 37°C, TrypLE Express was inactivated by dilution with DMEM/F12. Organoid containg solution was centrifuged at 200×*g* for 5 min and organoids were re-seeded in Matrigel at a dilution of 1:2–3.

### 2.3. Bovine GCO immunocytochemistry

GCOs cultured in Matrigel were treated with 500 μL of 4% paraformaldehyde (PFA, Thermo Scientific) at room temperature for 15 min. Following the treatment with 0.3% Triton X-100 (Thermo Scientific) and blocking with 2% bovine serum albumin (BSA, Cytiva), primary antibodies were applied to the organoids. To visualize E-cadherin, EpCAM, MUC5AC, and SOX9, monoclonal anti-mouse E-cadherin antibody (36/E-cadherin, BD Biosciences), polyclonal anti-rabbit EpCAM antibody (ab71916, Abcam), monoclonal anti-mouse MUC5AC antibody (45 M1, Thermo Fisher Scientific), and monoclonal anti-rabbit SOX9 antibody (EPR14335-78, Abcam) were used as primary antibodies. Then, the organoids were washed with PBS twice, and incubated with secondary antibodies (Anti-Mouse IgG H&L labeled with DyLight 488 and Alexa Fluor 555, and Anti-Rabbit IgG H&L labeled with DyLight 488 and Alexa Fluor 555). After washing, nuclei and F-actin were stained using 4′,6-diamidino-2-phenylindole dihydrochloride (DAPI) (Thermo Fisher Scientific) and Alexa Fluor 647 Phalloidin (Thermo Fisher Scientific), respectively. Then, washed organoids were suspended in Prolong Gold Antifade reagent (Thermo Fisher Scientific) and then mounted on a glass bottom dish (Matsunami). Stained organoids were imaged using white light point scanning confocal microscope (SP8-X, Leica). Acquired images were processed by LAS X (Leica).

### 2.4. Proliferation assay

To detect proliferative cells in bovine GCOs, 5-ethynyl-2-deoxyuridine (EdU) assay was conducted based on the manufacture’s protocol (Thermo Fisher Scientific). Briefly, bovine GCOs were preincubated with 10 μM of EdU solution for 3 h and fixed using 4% PFA (Thermo Scientific). After the fixation, organoids were permeabilized using 0.3% Triton X-100 (Thermo Scientific) and stained using EdU staining solution. Then, the nuclei were stained by DAPI (Thermo Fisher Scientific) for 30 min and stained organoids were mounted using Prolong Gold Antifade reagent (Thermo Fisher Scientific). The EdU (+) cells within the organoids were imaged using white light point scanning confocal microscope (SP8-X, Leica). Acquired images were processed by LAS X (Leica).

### 2.5. Organoid swelling assay

In order to confirm whether physiologically functional ion channels are at work in GCOs, we investigated the function of CFTR, one of the ion channels that is mainly expressed in epithelial cells. Forskolin-induced swelling assay was performed to confirm the functionality of CFTR (25). Bovine GCOs were seeded into 48-well culture plate with 30 μL of Matrigel and 300 μL of organoid culture medium. After the maturation of organoids, organoids were divided into three groups. The carrier control group was treated with dimethyl sulfoxide (DMSO, Sigma-Aldrich). The forskolin group was treated with fresh medium containing 10 μM Forskolin (Thermo Scientific). In order to specify whether the swelling was caused by activation of CFTR, the negative control group of organoids were pre-incubated with 50 μM CFTR(inh)-172 (Sigma-Aldrich), selective inhibitor of CFTR, prior to forskolin stimulation. Bright-field images of organoids were obtained using phase-contrast microscopy (DMi1, Leica) before stimulation and 1 and 2 h. The surface area of organoids was quantified using ImageJ (FIJI, http://fiji.sc/Fiji) (26). The average percent change of the surface area was calculated and compared in each condition. This experiment was conducted with three biological and three technical replicates. At each time, randomly selected five fields in each well and a minimum of 10 organoids were evaluated.

### 2.6. Statistical analyses

Quantitative data were analyzed using R Studio v1.4.1717 (RStudio). Figure was generated using GraphPad Prism 9.5.1 (GraphPad Software). For Forskolin-induced swelling assay, the changes in area were compared among three conditions using Kruskal-Wallis test and Dunn test. The results were presented as mean ± standard error of the mean. A *p* value was processed using Bonferroni correction and *p* < 0.05 was judged significant.

## 3. Results

### 3.1. Establishment of bovine GCOs

**Figure 1:**
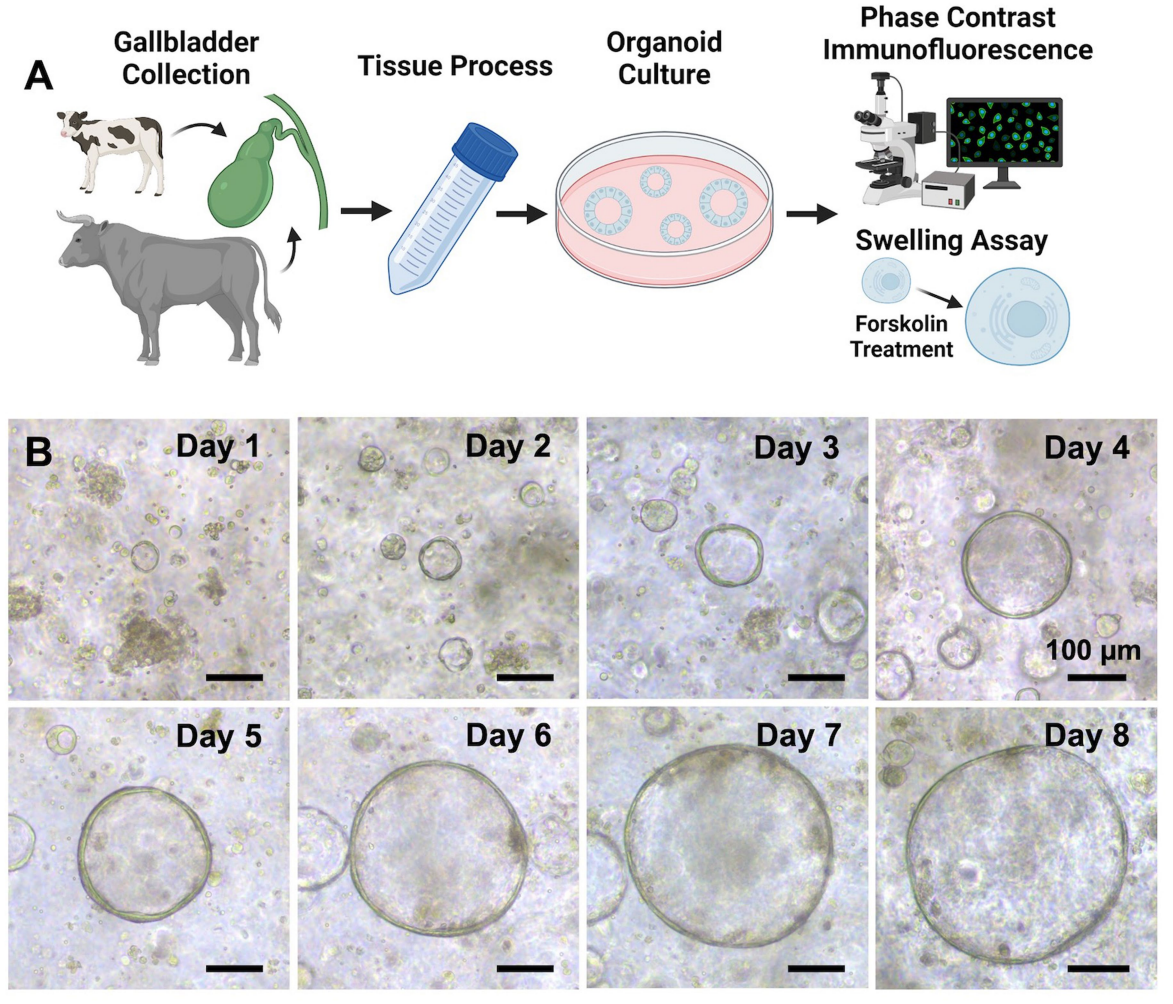
Establishment of organoids derived from bovine healthy gallbladder. **(A)** A schematic of our experimental design. Normal gallbladder tissues were obtained from calves and cattle that were euthanized or slaughtered humanely (donor information for the tissues used in the study is presented in Supplementary Table S1), and the processing method is detailed in the Methods section. After establishing the organoids, various techniques such as phase contrast imaging, immunofluorescence staining, and confocal imaging were employed to characterize them. Additionally, a forskolin-induced swelling assay was performed to further assess the functionality of cystic fibrosis transmembrane conductance regulator (CFTR) within the organoids. Created with BioRender.com. **(B)** A growth profile of the GCOs isolated from a donor. Representative phase contrast brightfield microscopy images of organoids were taken at days 0–8. Scale bar = 100 μm. Created with BioRender.com.

We generated 3D GCOs from isolated epithelial cells taken from the gallbladder tissue of four healthy calves and five healthy adult cattle following humane euthanasia using gunshot method (Figure 1A). The signalment of each donor is summarized in Supplementary Table S2. The generation efficiency was 77.8%, which is comparable to what has been reported for intestinal organoids (27). Two of the donors failed to grow; one was contaminated by bacteria, and the other organoid line failed to develop, likely due to autolysis during sample transportation.

Bovine GCOs formed within 2–3 days of dissociating the gallbladder epithelial cells. They developed a spherical structure with a central lumen surrounded by a layer of epithelial cells (Figure 1B), as previously reported in healthy humans (11), mice (28), and pigs (29). These organoids were successfully expanded for an average of four passages and maintained stable cultures for 1–2 months.

### 3.2. Characterization of bovine GCOs

**Figure 2:**
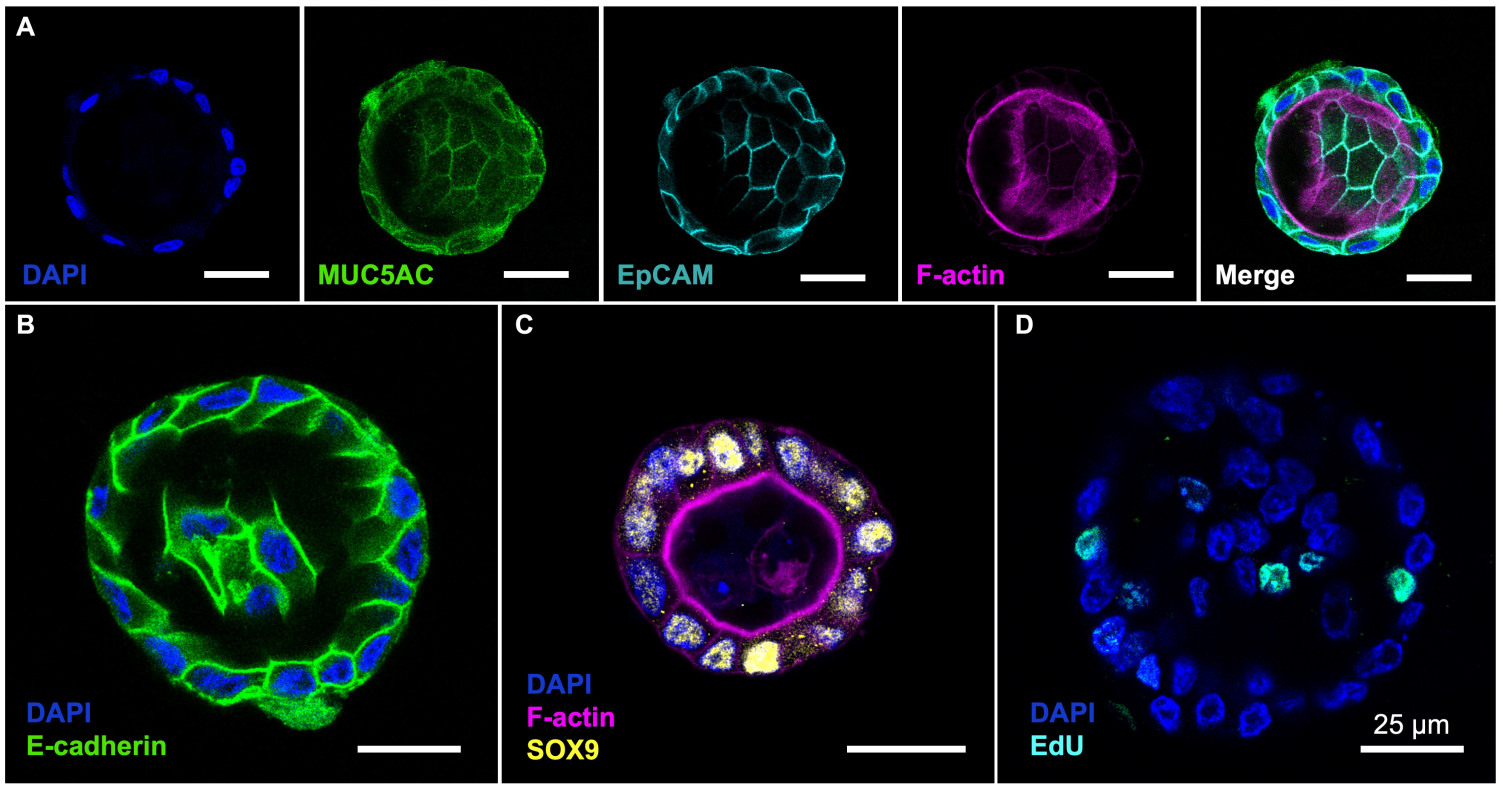
(Pattern A) Characterization of bovine GCOs. The bovine GCOs on day 4 were used to visualize the markers highlighting the characteristics of the cells within normal gallbladder. **(A)** Immunofluorescence (IF) staining on GCOs confirms expression of MUC5AC (Green) and EpCAM (Cyan) with a counterstaining of F-actin (Magenta) and nuclei (Blue). The population of E-cadherin (**(B)**; Green), stem cells (**(C)**; SOX9, Yellow), and proliferative cells (**(D)**; EdU, Cyan) were also visualized by using IF staining. As a counterstaining, F-actin (Magenta for **(C)**), or nuclei (Blue for **(B–D)**) were displayed. Scale bar = 25 μm. (Pattern B) Characterization of bovine GCOs. The bovine GCOs on day 4 were used to visualize the markers highlighting the characteristics of the cells within normal gallbladder. **(A)** Immunofluorescence (IF) staining on GCOs confirms expression of MUC5AC (Green) and EpCAM (Cyan) with a counterstaining of F-actin (Magenta) and nuclei (Blue). The population of E-cadherin (**(B)**; Green), stem cells (**(C)**; SOX9, Yellow), and proliferative cells (**(D)**; EdU, Cyan) were also visualized by using IF staining. As a counterstaining, F-actin (Magenta for **(C)**), or nuclei (Blue for **(B–D)**) were displayed. Scale bar = 25 μm.

Using MU5AC staining, we confirmed that all cells in the bovine GCOs produced MUC5AC, a gel-forming mucin commonly found in the gallbladder (30) (Figure 2A). These organoids were composed of EpCAM-positive cells, confirming that these cells are epithelial cells. The GCOs showed clear E-cadherin staining at the cell borders, indicating that the tight formation of junctional proteins at the cell borders (Figure 2B). We also performed SOX9 staining on the organoids, which is one of the stem cell markers (28), and observed distinct nuclear staining (Figure 2C). This finding confirms the presence of SOX9-positive cells in the bovine GCOs and suggests the retention of stem cell characteristics in these cells. Additionally, the EdU assay confirmed the presence of actively proliferating cells in the bovine GCOs, indicating ongoing cell division (Figure 2D).

### 3.3. Forskolin-induced swelling assay

**Figure 3:**
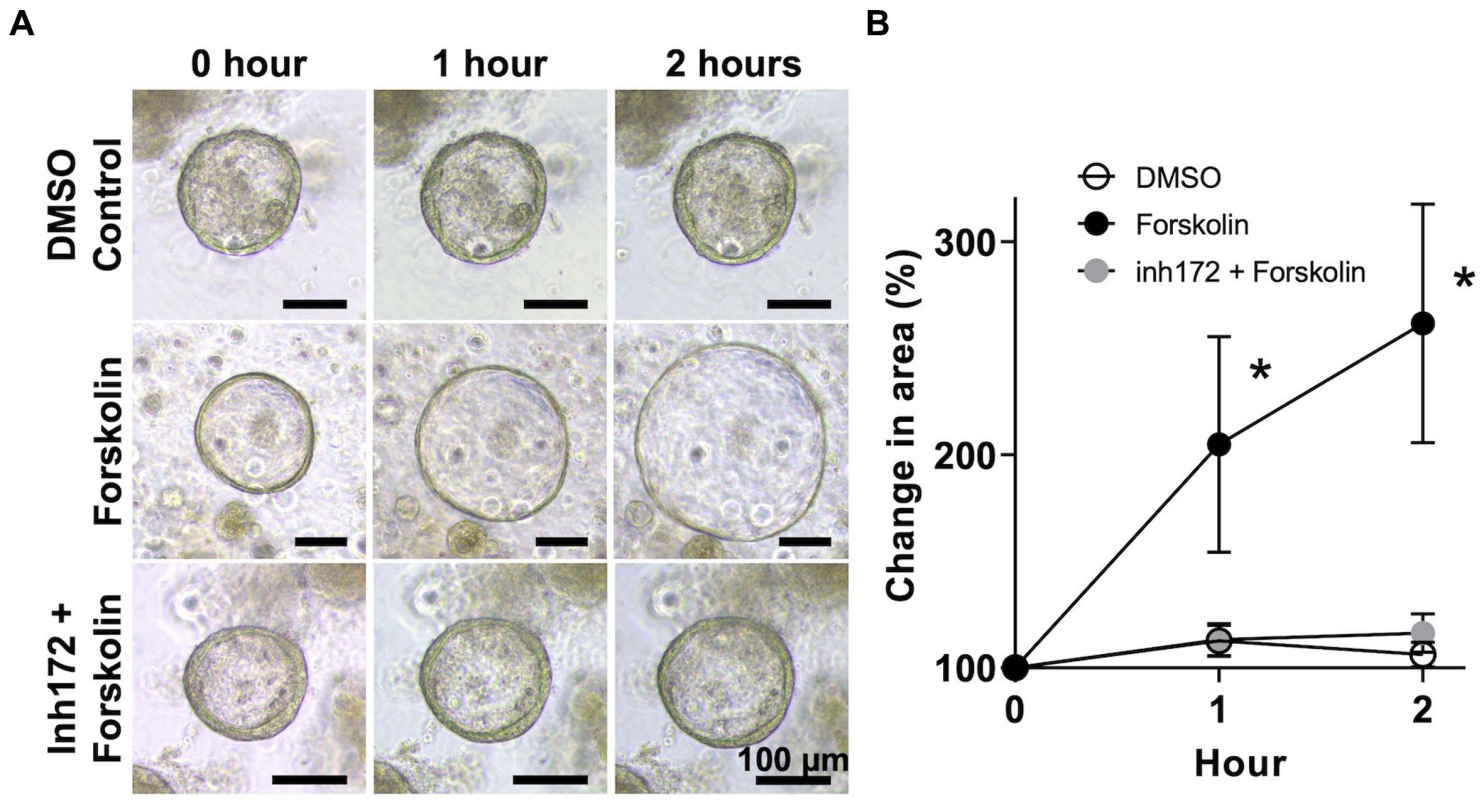
Swelling assay of bovine GCOs for CFTR function. **(A)** After organoids reaching matured on day 6, bovine GCOs were incubated in media containing vehicle control (DMSO) (top row) or 10 μM forskolin (second row). The third group of organoids was treated with 50 μM CFTR(inh)-172 prior to the forskolin treatment (bottom row). Representative images of organoids were taken at 0, 1, and 2 h following the treatment, at ×10 magnification. Scale bar = 100 μm. **(B)** The profile of % change in the area of organoids from randomly selected five fields per condition a minimum of 10 organoids (closed circle; forskolin, open circle; DMSO control, grey circle; CFTR(inh)-172) as determined by ImageJ among three biological replicates. In each biological replicate, three technical replicates were performed. Error bars indicate SEM. *p < 0.01.

Next, to confirm the function of CFTR, we performed an organoid swelling assay using forskolin, a cyclic adenosine monophosphate (cAMP) agonist that activates CFTR in bovine GCOs (25) (Figure 3A). CFTR is a chloride ion channel expressed in epithelial cells that secretes water from the apical side of the cells when the intracellular cAMP concentration increases. If CFTR is functional, this water secretion causes the organoids to swell (25). In the DMSO control group, the size of the organoids increased slightly after exposure to the solvent, with a mean size of 112.6 ± 6.8% and 106.3 ± 5.4% larger from 0 h after 1 h and 2 h, respectively. These results indicate that exposure to DMSO alone cannot lead to significant increase in the size of the GCOs. In the forskolin treatment group, the size of the organoids increased significantly after exposure to 10 μM of forskolin, with a mean size of 204.8 ± 47.7% (*p* < 0.01) and 261.6 ± 52.7% (*p* < 0.01) larger compared to the size at 0 h after 1 h and 2 h, respectively. These results indicate that exposure to forskolin alone can lead to a significant increase in the size of the GCOs. Furthermore, the observed size increase of bovine GCOs following forskolin treatment was diminished when the organoids were pre-treated with CFTR(inh)-172, a selective inhibitor of CFTR (Figure 3B).

## 4. Discussion

This proof-of-concept study reports the successful establishment and characterization of bovine GCOs that preserve key characteristics of the gallbladder *in vivo*. We generated a total of seven lines of GCOs from calves and cattle and confirmed their expression of stem cell characteristics and proliferative properties. Notably, our results demonstrate specific and functional CFTR activity in bovine GCOs. To the best of our knowledge, this is the first study to report the stable culture of healthy GCOs from calves and cattle. Overall, our findings provide a valuable tool for studying the physiology and pathophysiology of the bovine gallbladder *in vitro*.

The scarcity of normal human gallbladder epithelium has been a significant constraint in investigating its physiological and pathological properties. However, this limitation has been mitigated by the development of liver transplantation programs (31), which have made normal gallbladder tissue more accessible. Additionally, animal models have been extensively utilized as an alternative approach to overcome the lack of availability of normal human tissue. Notably, the remarkable similarity in the composition of bile, as well as the mechanisms of electrolyte transport and hydrogen ion secretion, between bovine and human species, is particularly noteworthy and carries significant value in the study of gallbladder physiology (13, 14). Moreover, research on the ion transport and electrical properties of the gallbladder epithelium in animal models can help to identify potential targets for drug development to treat these conditions.

The presence of persistent gallbladder infections with *E. coli* O157:H7 and *Salmonella* that can affect both human and animal populations can have significant public health implications (32, 33). Investigation of bacterial colonization utilizing bovine GCOs can offer valuable insights into the pathogenesis of infectious diseases in humans, as well as potential therapies to prevent or treat such infections. Recent advancements in 3D organoid culture applied to 2D monolayer culture represent a breakthrough in the study of host-pathogen interactions (34), particularly for zoonotic pathogens that are transmitted through the consumption of contaminated food or contact with infected animals. Similar application can be made with bovine GCOs to investigate the mechanisms by which these pathogens colonize and persist in the bovine gallbladder. This could lead to the development of more effective control measures to reduce the risk of human exposure to these pathogens.

In this study, modified medium was used to generate normal GCOs, and a success rate of 77.8% was achieved. However, initial attempts using our current medium without HGF, FGF-10, and dexamethasone resulted in low efficiency of organoid generation (data not shown). Our current medium was modified from Yuan et al. (11), and we removed Prostaglandin E2 (PGE2), Forskolin, and Insulin-like growth factor (IGF), which were not commonly included in the media described in other studies (12, 28, 35). Our medium contains Noggin, R-spondin-1, EGF, FGF-10, and HGF, which are all the growth factors included in the media listed in Supplementary Table S1 (28, 35). Despite the minor modifications we made to the medium, our results suggest that it was effective in promoting the growth and maintenance of GCOs. Moreover, we tried another medium, William’s E medium based complete medium (29), but organoids were not developed from single cells. The failure of the culture using modified medium in this study was attributed to several factors, including contamination of the samples and autolysis of gallbladder tissues during transportation. The latter issue led to a reduced number of available epithelial cells for organoid generation and decreased cell vitality, ultimately contributing to the low generation rate. Furthermore, the number of passages of GCOs could not continue as long as reported in humans and mice (11, 28). We suspect the support of proliferation of stem cells was not enough in bovine GCOs. Similar situations have been reported in other animal organoids (7, 36). Specifically, several different compositions of media reported in humans and mice were tried to grow feline intestinal organoids, but growth stopped without reaching the passages as reported in other animal species (36). To date, the cause of the cessation of its proliferation is unclear, however, it is thought that a species-specific factor may be necessary to promote the proliferation of organoids. In other case, human pancreatic organoids stopped the proliferation in early passage, though mice pancreatic organoids can proliferate in the same condition more than 30 passages (7). Examination of the medium conditions revealed that the nutrient composition requirements of mouse and human organoids are very different. To improve the efficiency and maintenance of organoids, the generation protocol and culture conditions should be further optimized in future studies (7).

In conclusion, our proof-of-concept study demonstrates that the bovine GCOs retained key characteristics of normal gallbladder tissue as well as replication property, and provides a useful model for studying gallbladder function. Understanding the role of ion channels in gallbladder function and dysfunction can help in developing new therapeutic strategies for these conditions. The development of bovine GCOs opens up new possibilities for studying the function of human gallbladder, advancing our understanding of basic physiology and disease mechanisms across different species.

## Data availability statement

The original contributions presented in the study are included in the article/Supplementary files, further inquiries can be directed to the corresponding author.

## Ethics statement

The animal study was reviewed and approved by Washington State University Institutional Animal Care and Use Committee. Written informed consent was obtained from the owners for the participation of their animals in this study.

## Author contributions

IN and YA designed the experiments, wrote and edited the manuscript, tables, and figures. IN performed the experiments. All authors contributed to the article and approved the submitted version.

## Funding

This work was supported in part by the Office of the Director, National Institutes of Health (K01OD030515 and R21OD031903 to YA) and Japan Society for the Promotion of Science Overseas Challenge Program for Young Researchers (202280196 to IN).

## Conflict of interest

The authors declare that the research was conducted in the absence of any commercial or financial relationships that could be construed as a potential conflict of interest.

## Publisher’s note

All claims expressed in this article are solely those of the authors and do not necessarily represent those of their affiliated organizations, or those of the publisher, the editors and the reviewers. Any product that may be evaluated in this article, or claim that may be made by its manufacturer, is not guaranteed or endorsed by the publisher.
