# Supplementary material for: Ion channel function in translational bovine gallbladder cholangiocyte organoids: establishment and characterization of a novel model system
Source: Front Vet Sci. 2023 May 26;10:1179836. doi: 10.3389/fvets.2023.1179836 (PMC10250713; doi:10.3389/fvets.2023.1179836)
Supplement: Supplementary file 1 [file Data_Sheet_1.docx]

Supplementary Material

# Supplementary Data

**
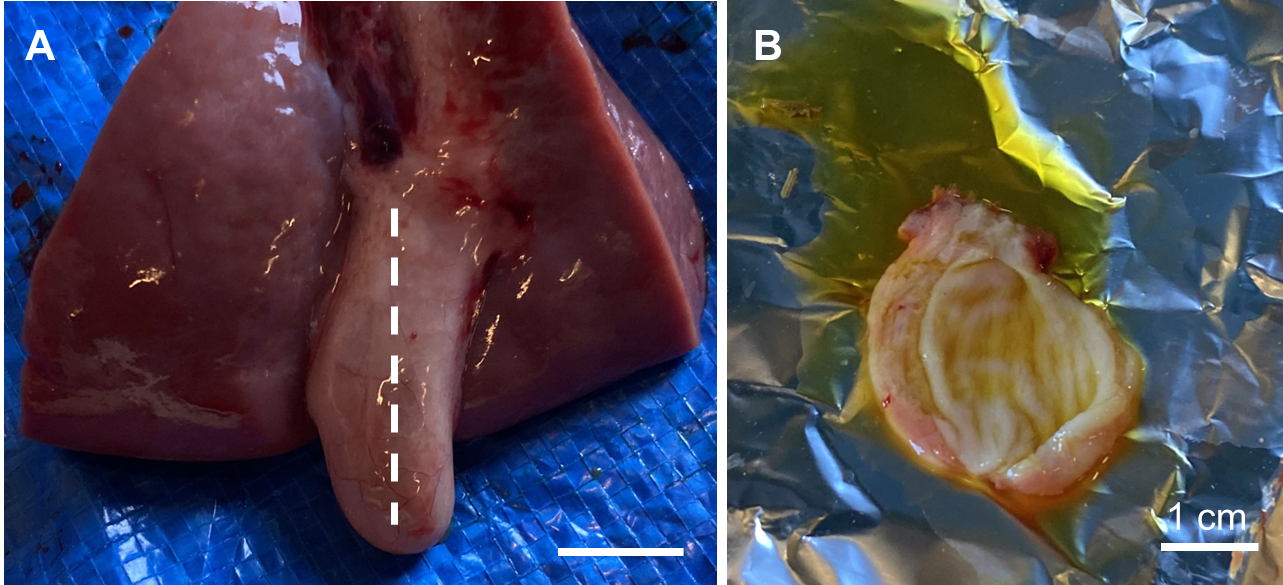
**

**Supplementary Figure 1. Gallbladder tissue isolation from calves and cattle for organoid generation.** (A) Immediately following the humane euthanasia or slaughtering, the gallbladder was isolated using sterile surgical scissors (A) and opened longitudinally (B). The white dotted line demonstrating the usual incision to open the gallbladder. Subsequently, a tissue sample was placed in Dulbecco’s phosphate-buffered saline as described in the Methods section**.**

**Supplementary Table 1.** Comparison of medium used for Gallbladder Cholangiocyte Organoid culture with previous reports.

**Supplementary Table 2.** A summary table of the donor information with sample name, breed, sex, age, sex, and major cause of cell death or failure of organoid establishment.
